# Adaptive immunity in the pathogenesis and treatments of Parkinson’s disease

**DOI:** 10.1515/nipt-2025-0008

**Published:** 2025-06-20

**Authors:** Xiaoqing Du, Samia Akter, Davina B. Oludipe, Susmita Sil, Chen Zhang, Howard E. Gendelman, R. Lee Mosley

**Affiliations:** Department of Pharmacology and Experimental Neuroscience, College of Medicine, 12284 University of Nebraska Medical Center, Omaha, NE, USA

**Keywords:** Parkinson’s disease, adaptive immunity, alpha-synuclein

## Abstract

Neuroimmunity drives the pathophysiology of Parkinson’s disease (PD). This disease affects both the central and peripheral nervous systems. The immune system is engaged through the progressive accumulation of alpha-synuclein (α-syn), a driver of immunity and a pathological hallmark of PD. Consequent α-syn-induced immune activation leads to neuronal damage. This leads not only to the activation of microglia within the central nervous system, but also to the recruitment and activation of peripheral immune cells that infiltrate the brain and contribute to a widespread immune response. Moreover, PD-associated genes and risk factors have been increasingly recognized as essential regulators of immune functions. This review summarizes the current understanding of adaptive immunity in PD and explores emerging immunomodulatory strategies that may inform future therapeutic development.

## Introduction

**Figure 1:**
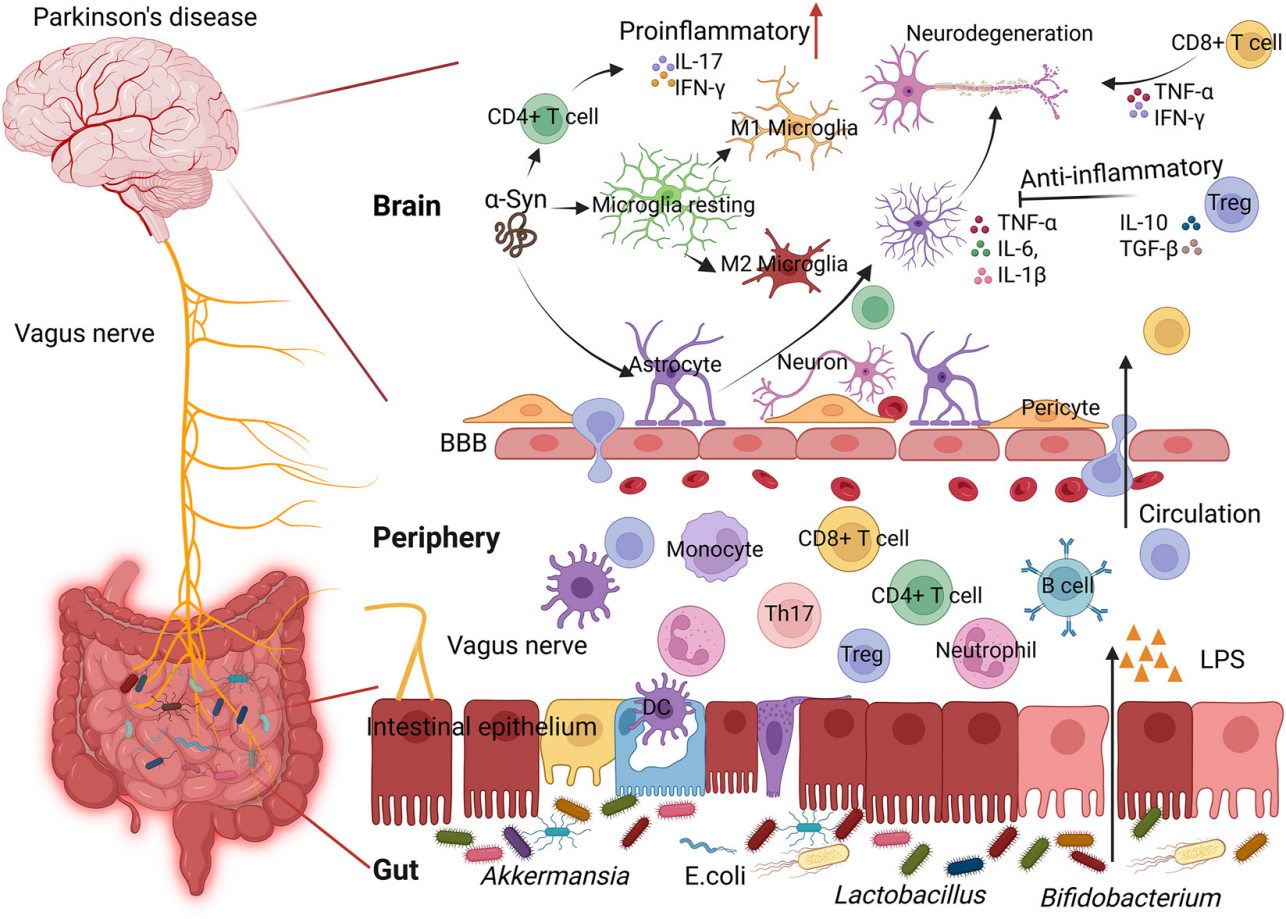
Neuroinflammation and adaptive immune response in PD. Exposure of modified and aggregated α-synuclein (α-syn) in the brain activates microglia that secrete neurotoxic proinflammatory cytokines such as TNF-α, IL-6 and IL-1β. Modified α-syn and inflammatory mediators drain to peripheral immune tissues to activate antigen presenting cells that lead to induction of α-syn-specific CD4+ and CD8+ T cells that differentiate to specific effector T cells (Teffs), such as Th1, Th17, and Tc, extravasate to foci of brain inflammation, and release proinflammatory cytokines such as IFN-γ, IL-17, and TNF-α that exacerbate microglial activation, inflammation, and neurodegeneration. These responses also induce astrocyte activation, resulting in the production of additional inflammatory mediators including TNF-α, IL-6, and IL-1β, thereby contributing to neuronal degeneration. Regulatory T cells (Tregs) infiltrating the brain counteract neuroinflammation by suppressing activated astrocytes through the secretion of IL-10 and TGF-β. According to the “gut-first” hypothesis, dysbiosis of the intestinal microbiota disrupts gut metabolic activity and barrier integrity, leading to increased levels of microbial products such as lipopolysaccharide (LPS). Elevated levels of LPS can translocate into systemic circulation, promoting peripheral immune activation. Microbial dysbiosis and breech of the gut barrier along the gut-brain axis may contribute to α-syn misfolding and aggregation, central inflammation via immune cell trafficking, and spreading and transmission via vagus nerve.

Parkinson’s disease (PD) is a chronic, progressive neurodegenerative disorder primarily defined by the loss of dopaminergic neurons in the substantia nigra pars compacta (SNpc) and the accumulation of α-synuclein aggregates, forming Lewy bodies. While many studies have focused on mitochondrial dysfunction, oxidative stress, and protein clearance deficits, growing evidence suggests that immune dysregulation significantly contributes to disease onset and progression (Figure 1) [1], 2]. Recent findings have indicated both innate and adaptive immune responses in PD [3]. T lymphocytes, both CD4+ and CD8+ T cells, have been observed in the substantia nigra (SN) of patients with PD and may play a direct role in neuronal injury. CD8+ T cell infiltration has been observed early in the disease course, even before detectable α-synuclein (α-syn) aggregation and dopaminergic neuron loss and express cytotoxic molecules such as granzyme A/B and IFN-γ, suggesting that adaptive immune responses may contribute to the initiation or progression of neurodegeneration [4]. Supporting this idea, T cells from PD patients have been shown to recognize specific α-syn-derived epitopes presented by major histocompatibility complex (MHC) molecules, implicating antigen-specific adaptive immune responses in PD pathogenesis [5]. Although the precise triggers remain unclear, these findings suggest a potential link between α-syn pathology and T-cell activation. Immune alterations have also been reported at the systemic level. Peripheral T and B cells in patients with PD show clonal expansion and are skewed toward effector and memory phenotypes, indicating that adaptive immune responses are not confined to the central nervous system [6]. Moreover, elevated neutrophil-to-lymphocyte ratios have been observed years before PD diagnosis, suggesting that immune dysregulation may occur early in the disease course [6]. The role of B cells in PD is poorly understood and remains unclear. Some studies have reported reduced peripheral B-cell counts in patients with PD, although the findings are inconsistent [1]. Evidence of IgG deposition on dopaminergic neurons and the expression of Fcγ receptors on activated microglia suggests that humoral immunity may contribute to neuroinflammation. Additionally, autoantibodies against α-syn have been detected in the serum and cerebrospinal fluid of patients with PD, and their levels appear to correlate with disease activity, raising the possibility that these antibodies could serve as potential biomarkers. Together, this research suggests that the adaptive immune system is not merely a bystander in PD but may actively contribute to disease development. This review will provide a summary of classical PD mechanisms and explore how T- and B-cell activity intersect with neurodegeneration, with a focus on emerging immunomodulatory strategies targeting adaptive immunity.

## Pathogenesis and pathophysiology

PD is a progressive neurodegenerative disorder characterized by the loss of dopaminergic neurons in the SNpc and accumulation of misfolded α-syn aggregates, forming Lewy bodies. Its pathogenesis involves interconnected mechanisms, including α-syn aggregation, mitochondrial dysfunction, oxidative stress, impaired protein clearance, genetic mutations, and neuroinflammation. α-Syn, for which its aggregation is one of the hallmarks of PD and related synucleinopathies, is a 140-amino acid protein highly expressed in neurons, especially at synaptic terminals, where it regulates neurotransmitter release and vesicle trafficking [7]. Under physiological conditions, α-syn interacts with negatively charged lipids and adopts a membrane-bound α-helical structure [7], 8]. However, both unfolded and membrane-bound forms can be converted into β-sheet-rich amyloid structures, leading to oligomers, protofibrils, and Lewy bodies [9], [10], [11]. These oligomers are particularly neurotoxic and possess seeding potential that spreads misfolding across brain regions, impairing membranes, including dopaminergic vesicles and mitochondria, leading to cell death [12], 13]. Misfolding and aggregation are mediated by pathogenic mutations in the *SNCA* gene, as well as post-translational modifications such as serine 129 phosphorylation, ubiquitination, nitration, and truncation [8], [14], [15], [16]. Misfolded α-syn can spread from cell to cell, serving as a template that induces misfolding of native α-syn and causes synaptic dysfunction and neuronal injury, thereby propagating the disease with progressive loss of dopaminergic neurons in the SNpc and impaired motor coordination [17], 18]. Misfolded or aggregated α-syn can stimulate microglia and peripheral monocytes with an intensity of immune activation that varies according to their structural conformation. This innate immune activation may subsequently facilitate the recruitment and engagement of adaptive immune responses, possibly through antigen presentation and cytokine-mediated signaling [10]. Mitochondrial dysfunction, particularly complex inhibition, leads to the accumulation of reactive oxygen species (ROS), oxidative damage, and neuronal apoptosis [19]. When α-syn accumulates in the mitochondria, it contributes to complex I dysfunction, impairing electron transport and ATP synthesis, increasing reactive oxygen species (ROS), and oxidative damage. This mitochondrial stress may amplify α-syn pathology, forming a vicious cycle that drives neuronal death and PD progression, further impairs mitochondrial function, and creates a cycle of cellular stress [19], 20]. Damaged mitochondria release damage-associated molecular patterns (DAMPs), such as mitochondrial DNA (mtDNA), cardiolipin, and cytochrome c, which activate innate immune receptors such as toll-like receptors (TLRs) and the stimulator of interferon genes (STING) pathway, particularly in microglia [21]. These mitochondrial signals can also prime antigen-presenting cells, such as dendritic cells and microglia, possibly leading to the downstream activation of adaptive immune responses.

In addition, the clearance of unwanted and damaged proteins plays a critical role in PD pathogenesis [22]. Two major proteostasis systems, ubiquitin-proteasome system (UPS) and autophagy-lysosome pathway (ALP), are responsible for degrading damaged proteins [8], 22]. The UPS mainly clears soluble misfolded proteins via ubiquitin tagging and proteasomal guidance [23], while ALP handles insoluble aggregates through macroautophagy, microautophagy, and chaperone-mediated autophagy [24]. Dysfunction of either pathway promotes toxic α-syn accumulation. Mutations in PD-related genes, such as *SNCA*, *LRRK2*, *PINK1*, *PRKN*, *GBA*, and *DJ-1*, and most likely environmental exposures, contribute to disease onset by impairing mitochondrial homeostasis, protein degradation, and immune regulation. Notably, mutations in *SNCA*, *LRRK2*, and *PRKN* have been implicated in familial PD and linked to abnormal immune activation and enhanced neuroinflammatory responses [25], 26]. This pathological process leads to loss of dopaminergic neurons.

## Gene-driven adaptive immunity

Variants in the glucosylceramidase beta 1 (*GBA1*) gene, which encodes the lysosomal enzyme glucocerebrosidase (GCase) and is associated with Gaucher disease (GD), a lysosomal storage disorder involving immune system dysfunction [27], have also been identified in PD patients, suggesting a potential link between lysosomal dysfunction, immune dysregulation, and PD pathogenesis [28]. GD is a recessively inherited disease caused by *GBA1* variants. Notably, GD-naive *GBA1* variant carriers are susceptible to developing PD [29]. Several variants of *GBA1* are associated with the development of PD, including p.E326K, p.T369M, p.N370S, and p.L444P, with a concomitant decrease in glucocerebrosidase activity, the enzyme encoded by *GBA1*. Reduced glucocerebrosidase activity diminishes lysosomal degradation of α-syn [30]. The prevalence of *GBA1* variants varies across populations; for example, p.E326K has a 1–5% frequency in the European population, but is rare in Asians. Approximately 25 % of the PD risk is attributed to genetic variations [31], 32], and several of these genes modulate immune responses and neuroinflammation, including autosomal dominant variants such as *LRRK2 (PARK8)*, *SNCA (PARK1/PARK4)*, and *VPS35 (PARK17)*, and autosomal recessive mutations in genes such as *DJ-1 (PARK7)*, *PRKN (PARK2)*, *GBA*, and *PINK1 (PARK6)* [33], [34], [35].

Among these, *LRRK2* is one of the most significant genetic risk factors for both familial and sporadic PD, and is strongly associated with immune dysfunction. In addition to its known function in microglia, *LRRK2* is also found in adaptive immune cells, such as B and T lymphocytes. These cells regulate cytokine production, antigen processing, and signaling pathways involved in immune responses [35], 36]. Microglial activation in PD is further driven by α-syn aggregation, which engages receptors, such as TLR2, FcγRIIB, and CD36, triggering NF-κB signaling and nucleotide-binding oligomerization domain, leucine rich repeat and pyrin domain containing proteins-3 (NLRP3) inflammasome activation [37]. These pathways promote the release of IL-6, IL-1β, and tumor necrosis factor-α (TNF-α), thereby sustaining chronic neuroinflammation. Additionally, α-syn binding to FcγRIIB suppresses microglial phagocytosis via Src homology region 2 domain-containing phosphatase-1 (SHP-1) [38], 39], and uptake of α-syn fibrils involves Fyn kinase and CD36, amplifying IL-1β production [40]. Vacuolar protein sorting-associated protein 35 (VPS35), a critical component of the retromer complex, plays a role in endosomal sorting, autophagy, and mitochondrial function by sharing pathways with *LRRK2* [41]. Dysfunctional VPS35 disrupts autophagy-lysosomal pathways in dopaminergic neurons, cortical neurons, and microglia, leading to α-syn accumulation and impaired mitochondrial dynamics via interactions with dynamin-1-like protein-1 (DLP1) and mitochondrial E3 ubiquitin protein ligase 1 (MUL1) [42]. *DJ-1 (PARK7)* protects neurons, microglia, and astrocytes from oxidative stress by scavenging reactive oxygen species (ROS) and stabilizing nuclear factor erythroid 2-related factor 2 (NRF2), a key regulator of antioxidant responses. *DJ-1* deficiency results in pro-inflammatory microglial activation and increased TNF, IL-1β, and IL-6 release, exacerbating neurodegeneration [43]. Parkin (*PARK2*), an E3 ubiquitin ligase encoded by *PRKN*, works alongside *PINK1* to facilitate the degradation of damaged mitochondria [44], 45]. Mitochondrial depolarization leads to *PINK1* accumulation on the outer membrane, which recruits parkin to ubiquitinate dysfunctional proteins for degradation [46]. Overall, PD-associated genetic mutations not only lead to mitochondrial impairment and neuronal cell death but also contribute to chronic neuroinflammation and PD progression.

## Gut-brain axis

The gut-brain axis, a bidirectional communication network between the gastrointestinal (GI) system and CNS, plays a vital role in the pathogenesis of PD [47]. Extensive research has shown that alterations in the gut microbiota and intestinal permeability (“leaky gut”) contribute to neuroinflammation and PD progression (Figure 1) [48]. Gut microbiota, one of the most abundant and diverse microbial communities in the human body, has been associated with numerous diseases, including systemic inflammatory conditions. The gut represents a complex microecosystem inhabited by various microorganisms such as bacteria, parasites, archaea, fungi, and viruses [49]. Clinical studies have consistently reported gut microbiota alterations, known as dysbiosis, in patients with PD, with altered microbial profiles identified using high-throughput sequencing techniques [50]. A key finding in PD research is the increased abundance of bacteria traditionally regarded as beneficial, such as *Akkermansia, Lactobacillus*, and *Bifidobacterium*, which contribute to maintaining gut barrier integrity by enhancing the production of tight junction proteins [48], 51], 52]. However, disruption of the microbiome composition results in altered metabolic activities, gut barrier dysfunction, and compromised gut homeostasis [53], contributing to aberrant inflammatory responses that may accelerate neurodegeneration [54], 55]. In humans, the gut microbiota is a major source of lipopolysaccharide (LPS) [56]. LPS, an immunostimulatory component of Gram-negative bacterial cell walls, exhibits variable inflammatory and neurotoxic properties and can enter systemic circulation [53]. LPS activates TLR4-mediated inflammatory signaling in the gut, compromising intestinal barrier function. The resulting systemic inflammation has been shown to increase blood-brain barrier (BBB) permeability, allowing pro-inflammatory mediators to enter the CNS, disrupting Treg function, and intensifying neuroinflammation [57], [58], [59]. The “gut-first” hypothesis, proposed by Braak and colleagues, suggests that α-syn aggregates may originate in the enteric nervous system (ENS) and propagate to the CNS via the vagus nerve [60], [61], [62]. Additionally, studies have shown that gut luminal signals, particularly via glutamate metabolism, can be transmitted rapidly to glutamatergic neurons in the hippocampus through vagal pathways [63], suggesting a highly sensitive gut–brain communication mechanism. Braak’s theory suggests that gut dysbiosis, chronic inflammation, and microbial imbalance in the ENS may trigger α-syn misfolding, initiating PD pathology [64]. Microbial dysbiosis and chronic intestinal inflammation may contribute to α-syn misfolding in the gut. The innate immune system, including gut-resident macrophages and enteric endothelial cells, responds by releasing pro-inflammatory cytokines (e.g., IL-1β, IL-6, and TNF-α), which may enhance α-syn aggregation and propagation to the CNS [65]. Disruption of the BBB, potentially triggered by peripheral immune activation and microbial inflammation in the gut, facilitates the infiltration of adaptive immune cells such as T and B lymphocytes into the central nervous system [66], contributing to sustained neuroinflammation and the progression of Parkinson’s disease.

## Immune cell subsets in PD pathogenesis

### T effector cells (Teffs)

While T cells are sporadic in the CNS of healthy subjects, mostly in the choroid plexus or CSF, most T cells found in the parenchyma result from the infiltration of activated T cells from the periphery [66]. Both CD4+ and CD8+ T cells have been reported to be involved in neurodegeneration in patients with PD [67]. Additionally, increased frequencies of T cells with Teff phenotypes in patients with PD have been correlated with motor function severity [68]. The migration of peripheral T cells into the CNS occurs after T cell ligands on endothelial cells bridge integrins and selectins on T cells that capture lymphocytes and allow extravasation into the brain and injurious foci (Figure 1) [66]. Both CD4+ and CD8+ T cells extravasate into the CNS through the interaction of α4β1-integrin with activated T cells and vascular cell adhesion protein 1 (VCAM-1) on capillary endothelial cells [69]. CD4+ and CD8+ T cells that are permitted to extravasate typically have Teff phenotypes, defined by the function and cytokines expressed upon reactivation in the CNS. Reactivation occurs when the T-cell receptor (TCR) recognizes its cognate antigen presented by MHC I or II on APCs such as microglia or macrophages. This induces Teff to initiate its defensive effector function, that is, the expression of cytokines or killer programs. In turn, Teffs have direct and indirect associations with neurons, astrocytes, and other microglia within the CNS, with varied responses depending on Teff type.

### CD4+ T cells

In PD, CD4+ Teffs play a critical role in the adaptive immune response in the CNS by promoting neurodegeneration through proinflammatory cytokines and chemokines as well as the Fas/FasL pathway [70]. Inflammatory conditions are permissive to CD4+ T cell migration through the BBB [71], 72]. Once inside, activated CD4+ Teffs become re-activated by microbial or modified self-antigens released from degenerating neurons that have been processed and presented by microglia or other APCs [73]. Modified-self antigens are implicated in the induction of autoreactive CD4+ Teffs responses in PD and PD models, including nitrated and phosphorylated α-syn [74]. In one report, most CD4+ Teffs from patients with PD were reactive to phosphorylated α-syn and exhibited a rare phenotype that primarily expressed IFN-γ and IL-5 [5]. In mice treated with MPTP, peripheral T cells respond to nitrated α-syn but not native α-syn, and polarization of CD4+ Teffs from the immune system to nitrated α-syn yields type-1 T helper (Th1) Teffs that express IFN-γ and type-17 T helper (Th17) Teffs that express IL-17 [75]. Adoptive transfer of either Th1 or Th17 Teffs to MPTP mice exacerbates MPTP-induced microglial activation and dopaminergic lesions; however, Th17 Teffs significantly enhanced these effects. Interestingly, a meta-analysis of clinical studies confirmed the association between elevated Th17 Teffs levels and PD and found that the percentage of Th17 cells correlated with motor impairments in patients [76]. Teffs interactions with microglia and astrocytes, particularly via pro-inflammatory cytokines, exacerbate the inflammatory cascade [77], 78]. Thus, targeting the inflammatory pathways mediated by CD4+ T cells is a potential therapeutic strategy for slowing PD progression [79], 80].

### CD8+ T cells

CD8+ T cells play a significant role in CNS through their involvement in adaptive immunity and impact on neurological conditions. The primary responsibility of CD8+ T cells in the periphery and CNS is to kill infected cells by recognizing antigens presented by MHC I molecules. Upon encountering antigens presented by MHC I, CD8+ T cells differentiate into cytotoxic effector T cells (Tc), which are capable of releasing cytokines, such as TNF-α and IFN-γ, and cytotoxic molecules, such as granzymes and perforins, to induce apoptosis of target cells [81]. In the brain and elsewhere, CD8+ Tc cells are responsible for providing defense against virus-infected cells by attacking and destroying viral-infected cells such as neurons [82]. This cytotoxic function can also contribute to neuroinflammation and neuronal damage in conditions such as traumatic brain injury (TBI) and ischemic stroke, where CD8+ T cells infiltrate damaged brain tissue, exacerbating neuronal cell death [83], [84], [85].

In PD, CD8+ Teffs are found in both peripheral blood and cerebrospinal fluid, indicating their activation and possible involvement in the progression of these disorders [84], 86]. Interestingly, in patients with PD and animal models, both CD4+ and CD8+ T cells infiltrate the brain, with studies reporting a reduced CD4+/CD8+ ratio, suggesting a relative predominance of cytotoxic CD8+ T cells in neurodegenerative regions. In the brains of patients with early PD, robust infiltration of CD8+ T cells was found with little change in CD4+ T cells; however, later stages of PD presented milder infiltrates of CD8+ T cells, suggesting the potential contribution of CD8+ Teffs to pathological changes in PD [4]. Although, whether CD8+ Teffs recognize cognate antigen in PD is uncertain, the increased clonality of the TCR repertoire in PD patients suggests that antigen-specific CD8+ *Teff* responses triggered by CNS antigens lead to increased release of proinflammatory mediators, such as IFN-γ and TNF-α, as well as perforins and granzymes that contribute to chronic neuroinflammation and neurodegeneration [87]. However, whether CD8+ T-cell clonality is due to PD processes or from a constricting repertoire due to aging is unknown [88]. Additionally, in the context of neuroinflammation, CD8+ T cells have been shown to shift immune responses, potentially leading to demyelination and neurological impairments [89]. Thus, the presence of clonal CD8+ T cells, given their cytotoxic capabilities, underscore the possible role of CD8+ T cells in dopaminergic neurodegeneration and highlight their potential as therapeutic targets, however, their role in PD etiology and disease progression has yet to be determined.

### Regulatory T cells (Tregs)

Tregs play a crucial role in maintaining immune homeostasis and preventing chronic inflammation [79], 90]. Patients with PD exhibit reduced Treg activity compared to controls and show increased numbers of CD4+ Teffs that correlate with clinical motor scores. Exposure to immunizing levels of nitrated α-syn also reduces Treg activity in preclinical models [74]. Therefore, strategies to enhance Treg function are being explored to mitigate microglial reactivity, attenuate neuroinflammation, and enhance neuroprotection. Tregs have been shown to suppress pro-inflammatory α-syn activated microglia by expression of anti-inflammatory cytokines and by inducing microglia apoptosis via Fas/Fas ligand interactions [91], 92]. Tregs also reduce ROS production by activated microglia, and of considerable importance, Tregs also suppress activated Teffs and Teffs induction [93]. Moreover, Tregs have been shown to increase expression of glial cell derived neurotrofic factor (GDNF) and brain derived neurotrophic factor (BDNF) by astrocytes in animal models of PD [70], 92]. Immune modulatory agents such as granulocyte-macrophage colony-stimulating factor (GM-CSF) show potential to increase Treg numbers and functionality, thereby reducing neuroinflammatory processes and protecting neuronal integrity [79], 90], 94]. Phase 1 clinical trials of human GM-CSF (sargramostim, Leukine^®^) in PD patients demonstrated GM-CSF was safe and tolerated for up to 36 months, increased Treg frequencies and function, increased neuronal activity in cortical motor areas, and improved Unified PD Rating Scale part III (UPDRS III) scores [39], 80], 95]. Importantly, after 36 months of treatment and 1 month washout period, UPDRS III scores did not significantly increase from pre-treatment scores, in contrast to historical controls [80], 96]. Phase II clinical trials to assess the efficacy of GM-CSF are ongoing. Anti-CD3 monoclonal antibodies also promote Treg induction and function, which in turn induce apoptosis of activated Teffs and microglia via Fas/FasL interactions [79], 92], thus promoting non-mitogenic anti-CD3 antibodies as therapeutic strategies for inflammatory-mediated neurodegenerative disorders [97], 98]. Vasoactive intestinal peptide (VIP) and VIP receptor-2 (VIPR2) agonists also increase Treg frequency and function, and these agents and their induced Tregs are anti-inflammatory and neuroprotective in animal models of PD [99], 100]. Finally, treatment with low-dose IL-2 increases Treg number and function, and adoptive transfer of IL-2-induced Tregs is neuroprotective along the nigrostriatal axis in MPTP-treated mice, thus providing another promising Treg-inducing strategy for PD [101].

The primary role of Tregs is to maintain immunological tolerance, particularly in the context of controlling ongoing immune responses to prevent pathological outcomes. The main Treg attribute is the ability to attenuate inflammatory responses with anti-inflammatory cytokines such as IL-10 and TGF-β. Similarly, Tregs in the CNS play a critical role in maintaining immune homeostasis and controlling inflammation [102]. Brain-resident Tregs, characterized by markers such as CD69, are present in the CNS and help modulate neuroinflammatory responses by releasing anti-inflammatory molecules, such as IL-10 and amphiregulin, which inhibit astrogliosis and promote neuroprotection [70]. Under neuroinflammatory conditions, such as those found in multiple sclerosis and stroke, Tregs suppress autoreactive T cell responses and reduce damage by promoting remyelination and aiding in white matter repair [70]. In adaptive immunity, Treg interaction with CNS antigens leads to the release of anti-inflammatory factors that transform microglia towards a more neurotrophic M2 state, ultimately reducing the overall inflammatory response. Research suggests that peripheral Tregs might also contribute to CNS protection by not only mitigating systemic inflammation that ultimately influences CNS homeostasis but also by migrating to sites of neuronal injury and inflammation [103], [104], [105]. Notably, peripheral Tregs in patients with PD have diminished inhibitory activity compared to age- and environment-matched caregivers [68]. While the therapeutic potential of Tregs is promising in neurodegenerative disorders, such as PD and [70], 74], 79], 80], distinguishing the specific roles of circulating and resident Tregs remains a major challenge [102].

### B cells

In neurodegenerative diseases, B cells can contribute to CNS pathology through antibody production and antigen presentation, thus intensifying adaptive and innate functional capabilities [106]. B cells exacerbate neurodegeneration by releasing neurotoxic molecules, such as GM-CSF, IL-6, and TNF-α, which are injurious to neuronal structures and promote local inflammation in afflicted brain regions. Additionally, B cells support T cell activation through antigen presentation, although diminished from that of professional APCs such as DCs, microglia, and monocytes, sufficient presentation is provided to reinforce T cell-mediated inflammatory processes within the CNS and accelerate disease progression. While there is a general paucity of reports concerning B cells in PD [107], diminished levels of peripheral B cells have been documented in patients with PD and in some α-syn over-expression models [108]. Recently, single-cell RNA analyses of peripheral B cells from patients with PD showed evidence of decreased naïve B cells with increased levels and clonal expansion of memory B cells compared to age-matched controls [109]. As in most immune cells of PD patients, *LRRK2* expression is upregulated in B cells, suggesting a role of activated immune cells in PD patients [2]. Moreover, increased activity in stimulated B cells from *LRRK2* knockout mice suggested a regulatory function for *LRRK2* [110]. Although direct evidence of B-cell infiltration of neurodegenerative foci in PD has yet to be reported, sufficient evidence of antibodies to α-synuclein in blood and CSF abound [107], 111], 112], and IgG deposition has been found on dopaminergic neurons and associated with Lewy bodies in PD patients [1]. Moreover, antibodies against nitrated α-synuclein, but not native α-synuclein, are produced in mice intoxicated with MPTP [113]. Interestingly, stereotactic injection of IgG from patients with PD into the rat substantia nigra yielded significant increases in dopaminergic loss compared with rats treated with control IgG [114]. Though preliminary in scope, evidence is suggestive for putative roles for B cells or anti-CNS antibodies in PD.

## Immunotherapies

Like many other neurodegenerative disorders, interventional treatment of PD presents a significant challenge, due to the lack of therapies that can modify or arrest disease progression. Standard treatments, such as levodopa, replenish dopamine levels that address symptom management but do not address underlying pathological processes [90], 115]. Recent insights into the role of the immune system in PD have propelled the development of novel therapies that target immune pathways, thereby offering new hope for disease modification.

### α-syn

Both active and passive immunotherapeutic strategies have been developed to reduce the burden of α-syn aggregates [116]. Different approaches include (1) silencing the *SNCA* gene using small hairpin RNA and antisense oligonucleotides; (2) enhancing proteasomal activity and autophagy pathways using molecules such as the deubiquitinase inhibitor 1U1 and autophagy/lysosomal regulating transcription factors such as transcription factor EB (TFEB); and (3) inhibiting α-syn misfolding and aggregation [116]. Active immunization approaches involve vaccines designed to stimulate the immune system to produce antibodies against α-syn [116], 117]. Vaccines, such as AFFITOPE PD01A and PD03A, are mimotopes engineered to target specific epitopes of α-synuclein that promote α-syn clearance from the CNS [115], [116], [117], [118]. Vaccines can recognize and neutralize α-synuclein aggregates, potentially halting disease progression. Clinical trials are ongoing to evaluate the efficacy and safety [119].

In contrast, passive immunotherapy uses monoclonal antibodies (mAbs) that are parenterally administered and target native or modified α-syn [116], 117]. Prasinezumab (PRX002) is a prominent mAb that recognizes aa118-126 and was designed to bind and neutralize extracellular α-syn aggregates and prevent dissemination and toxicity [90], 115], 117], 118]. These and other mAbs have shown promise in preclinical models and are currently undergoing clinical trials to assess their therapeutic potential in humans [119]. Recently, antigen-recognizing antibody fragments such as intrabodies have shown potential for reducing the levels of misfolded proteins and providing neuroprotection [118]. An intrabody is a single-chain, antibody variable region fragment (scFv) that is designed to be expressed intracellularly and targets intracellular proteins. Thus, anti-α-syn intrabodies target α-syn intracellular aggregation and guide α-syn complexes toward proteasomal degradation.

### Growth factors

Gene therapy offers a promising avenue for delivering neurotrophic factors and genes directly to the affected brain regions using viral vectors to support the survival and function of dopaminergic neurons [115], 117]. An early clinical trial of direct intraputamen delivery of glial cell line-derived neurotrophic factor (GDNF) showed improved UPDRS III scores and ^18^F-dopamine storage [120]. This study provides the basis for several successive GDNF trials; however, these have yielded inconsistent results, and alternative delivery methods have been explored [121]. A clinical trial of the AAV2-GDNF construct delivered to the putamen showed no improvement in the post-administration UPDRS III scores after 18 months; however, ^18^F-DOPA uptake improved [122]. Thus, although repeated trials have exhibited inconsistent results, the use of GDNF in animal models and clinical trials remains an active area of investigation. Other neurotrophic factors under clinical evaluation with varying degrees of success using native or modified protein formulations or vector-formulated constructs include neurturin (NRTN), brain-derived neurotrophic factor (BDNF), armetin (ARTN), persephin (PSNP), cerebral dopamine neurotrophic factor (CDNF), mesencephalic astrocyte-derived neural factor (MANF), and nuclear receptor-related 1 protein or NR4A2 (Nurr1), a transcription factor for nuclear receptors that regulates dopaminergic neuron development and maintenance [123], [124], [125].

### CAR-Tregs

Based on strategies for cancer treatment, chimeric antigen receptor (CAR) T-cell platforms are being explored for neurodegenerative diseases [126]. CAR T cells utilize antigen recognition regions from variable domains of antibodies directed at the target of choice. The cDNA encoding the variable regions is constructed to encode a single-chain peptide of variable fragments (scFv) that retain antigen reactivity. The expression of this construct results in transmembrane orientation of an external antigen recognition site. Ligation of the cognate antigen and antigen recognition site initiates the appropriate signal cascade and interventional T-cell program. Similar to cancer therapeutics, several CAR T cell strategies utilize cytotoxic T cell platforms to initiate the killing of targets such as glial tumors or autoreactive B and T cells. However, this approach has several limitations for neurodegeneration, including the introduction of unregulated autoreactive cytotoxic and pro-inflammatory T-cells, which may potentially exacerbate neuroinflammation and disease progression. For neurodegenerative disorders with inflammatory components, a contrasting approach utilizes CAR-Tregs with CARs that target misfolded proteins associated with respective disorders, such as amyloid β (Aβ) for Alzheimer’s disease, superoxide dismutase 1 (SOD1) or Tar DNA binding protein-43 (TDP-43) for amyotrophic lateral sclerosis (ALS). For PD and synucleinopathies, modified proteins such as nitrated or phosphorylated α-syn could provide an appropriate recognition target for CAR. Strategies for this CAR-Treg platform would isolate patient Tregs, delete endogenous TCRs, transduce the CAR construct encoding the anti-modified α-syn scFv protein, and expand CAR-Tregs to be adoptively transferred to the patient. The underlying rationale for this strategy is based on the transformative nature of Tregs, which attenuate microglial- and Teff-mediated neuroinflammation, diminishes α-syn misfolding and aggregation, and provides neuroprotection to dopaminergic neurons along the nigrostriatal axis.

## Concluding Remarks

Abundant evidence from both human and animal studies supports the important role of the innate and adaptive immune systems in the pathogenesis and progression of PD. These immune responses change over time during disease progression. α-Syn, a central feature of PD pathology, not only forms toxic aggregates but also plays an active role in triggering and sustaining immune activation. It stimulates both innate and adaptive immune responses, promotes neuroinflammation, and contributes to neuronal damage. Because α-synuclein is recognized as an antigen by both microglia and peripheral monocytes, innate immune responses can be initiated in both the brain and the periphery. In addition to chronic inflammation mediated by microglia in the brain, changes in immune cell populations have also been observed in the peripheral blood of patients with PD, involving both innate and adaptive immune cells. Peripheral immune cells, such as T and B lymphocytes, have been shown to infiltrate the brain, further contributing to inflammation in the CNS. It is possible that an initial innate immune response to modified α-syn triggers a more sustained adaptive immune reaction that spreads damage to other parts of the brain. T cells require antigen presentation via MHC molecules, whereas B cells recognize antigens through their surface receptors. Once activated, adaptive immune cells may further promote inflammation and neurodegeneration. In the future, tracking inflammatory changes over time, along with peripheral immune profiling, microbiome analysis, α-syn measurements, and imaging, may help identify useful immune-based biomarkers for predicting disease risk and progression. PD is a complex disease that includes the multiple genetic variants, gut-brain interactions, immune cell changes, and clinical symptoms. A better understanding of how specific antigens activate the immune system, how immune cells change over time, and how the interaction between the brain and peripheral immune systems is essential for developing effective immunotherapies and identifying reliable biomarkers for PD treatment.
